# Application of intrahepatic fiducial markers in carbon ion radiotherapy for pancreatic cancer—Achieving higher precision treatment

**DOI:** 10.1002/acm2.70427

**Published:** 2025-12-19

**Authors:** Junya Nagata, Yohsuke Kusano, Masataka Komori, Yoshiki Takayama, Shogo Kurokawa, Atsushi Ito, Tadashi Kamada, Daisaku Yoshida, Shohei Kawashiro, Hiroyuki Katoh

**Affiliations:** ^1^ Radiological and Medical Laboratory Sciences Nagoya University Graduate School of Medicine Nagoya Aichi Japan; ^2^ Section of Medical Physics and Engineering Kanagawa Cancer Center Yokohama Kanagawa Japan; ^3^ Department of Radiation Oncology Kanagawa Cancer Center Yokohama Kanagawa Japan

**Keywords:** Carbon‐ion radiotherapy, in‐room CT, interfractional motion, marker matching, pancreatic cancer

## Abstract

**Background:**

Carbon‐ion radiotherapy (CIRT) delivers highly conformal tumor doses while sparing surrounding organs‐at‐risk (OAR). However, density variations along the beam path and interfractional target motion can reduce target coverage and expose OAR to high doses. These motions are typically corrected using target matching (TM) or marker matching (MM). Because the pancreas is close to the gastrointestinal tract and major vessels, fiducial marker implantation carries a risk of organ or vascular injury, and metal artifacts from markers can affect beam range calculations. TM with computed tomography (CT) also reduces treatment efficiency and prevents real‐time motion monitoring.

**Purpose:**

To improve pancreatic cancer CIRT by correcting interfractional target motion using fiducial markers implanted in hepatic segments 5 or 6.

**Methods:**

Twenty patients with implanted fiducial markers who underwent liver cancer CIRT at our hospital were selected. The effect of interfractional target motion correction by MM in pancreatic cancer CIRT was evaluated as simulation study. The initial planning CT and in‐room CT images were used for bone matching (BM), liver‐dome matching (LM), TM, and MM. Correlations between target motion and fiducial marker or liver‐dome motion were analyzed, and fractional dose distributions were calculated. Variations in clinical target volume coverage (ΔCTV V95%) and OAR doses were examined across strategies per fraction.

**Results:**

A strong correlation was observed between the motion of the target and the fiducial marker. The median ΔCTV V95% values were −4.68%, −3.97%, −1.07%, and −1.69% for BM, LM, TM, and MM, respectively. TM and MM significantly improved target coverage compared to BM and LM. OAR doses stayed within dose constraints for all methods.

**Conclusion:**

The proposed MM approach corrected interfractional target motion, achieving target coverage comparable to TM. In pancreatic cancer CIRT, managing gastrointestinal gas and body contour changes along with tumor motion may further optimize target coverage and improve clinical outcomes.

## 1 INTRODUCTION

Pancreatic cancer remains one of the most lethal malignancies worldwide, with approximately 509 000 cases reported in 2021.^1^ Early detection of pancreatic cancer remains challenging, and only 15%–20% of patients are eligible for curative surgical resection upon diagnosis.^2^ For unresectable, locally advanced cases, chemotherapy or chemoradiotherapy is recommended,^3^, ^4^ and radiotherapy using x‐rays or particle beams may also be employed.^5^, ^6^, ^7^

The carbon‐ion beam, a type of particle beam, is a high linear energy transfer (LET) radiation that delivers a sharp distal dose distribution due to the Bragg peak.^8^ This characteristic allows for reduced irradiation to adjacent organs‐at‐risk (OAR) and concentrated dose deposition within the tumor.^9^, ^10^ The relative biological effectiveness (RBE) of carbon‐ion is approximately 2–3 times greater than that of x‐rays, rendering them effective even against radioresistant tumors.^9^, ^11^

In carbon‐ion radiotherapy (CIRT) for pancreatic cancer, both gastrointestinal gas and interfractional target motion present significant challenges. Kusano et al. evaluated the impact of gastrointestinal gas on target coverage in scanning CIRT for pancreatic cancer and proposed a robust treatment planning method to account for gas variability.^12^ Kubota et al. reported a correlation between interfractional target motion and reduced target coverage in passive CIRT for pancreatic cancer and demonstrated improvements through target matching (TM) using an in‐room computed tomography (irCT) system installed within the treatment room.^13^ However, it is time‐consuming and reduces treatment throughput; moreover, it does not allow monitoring of intrafractional motion during irradiation.

Fiducial markers have been employed for patient setup using marker matching (MM) and for tumor localization during treatment.^14^, ^15^ These markers are projected on x‐ray images, enabling tumor‐based positioning, correction of interfractional motion, and improved target coverage.^15^, ^16^, ^17^ Additionally, intrafractional target motion can be monitored via x‐ray fluoroscopy or serial x‐ray imaging during irradiation. Despite these advantages, fiducial markers also present drawbacks. Fiducial marker placement is typically performed percutaneously under CT or ultrasound guidance.^18^, ^19^, ^20^ Due to the anatomical proximity of the pancreas to the gastrointestinal tract and major vessels, percutaneous puncture is technically challenging and associated with a high risk of organ or vascular injury.^21^ Moreover, metal artifacts from fiducial markers on planning CT images distort the CT values, leading to calculation errors.^22^ Because accurate CT values cannot be used to calculate the beam stop position, the spread‐out Bragg peak (SOBP) position may deviate from the tumor location during actual irradiation. This deviation may reduce the tumor dose.^23^ Therefore, fiducial marker placement directly into the pancreas should be avoided in pancreatic cancer CIRT.

This study aimed to correct interfractional target motion and improve target coverage in pancreatic cancer CIRT by implanting fiducial markers into hepatic segments 5 (S5) or 6 (S6). To the best of our knowledge, the proposed method of correcting interfractional target motion for pancreatic cancer CIRT using fiducial markers implanted in S5 or S6 has not been previously reported. As these hepatic regions are adjacent to the pancreas, interfractional target motion can be managed effectively without introducing artifacts into the irradiation field. Consequently, improvements in target coverage are anticipated.

## 2 MATERIALS AND METHODS

### 2.1 Simulation study

This study was designed as a simulation to evaluate the effectiveness of fiducial markers implanted in the liver for correcting interfractional target motion in pancreatic cancer CIRT. Because liver cancer data were used, gross tumor volume (GTV) for pancreatic cancer could not be defined. As shown in Section 2.3, the clinical target volume (CTV) was defined according to the Japan Carbon‐ion Radiation Oncology Study Group (J‐CROS) regulations.^23^ Furthermore, at our facility, liver CIRT is performed using marker matching (MM) and breath‐hold irradiation. Therefore, the initial planning CT (pCT) and irCT images used in this study were breath‐hold CT images obtained during maximum exhalation. For reference, in pancreatic CIRT at our facility, four‐dimensional CT (4D‐CT) scans are performed using a pCT scanner, and treatment plans are prepared using the iGTV method.^12^ A phase range comprising motion within 0.5 cm is determined during treatment planning, and gated irradiation is then used for treatment delivery.

### 2.2 Patient characteristics

**FIGURE 1:**
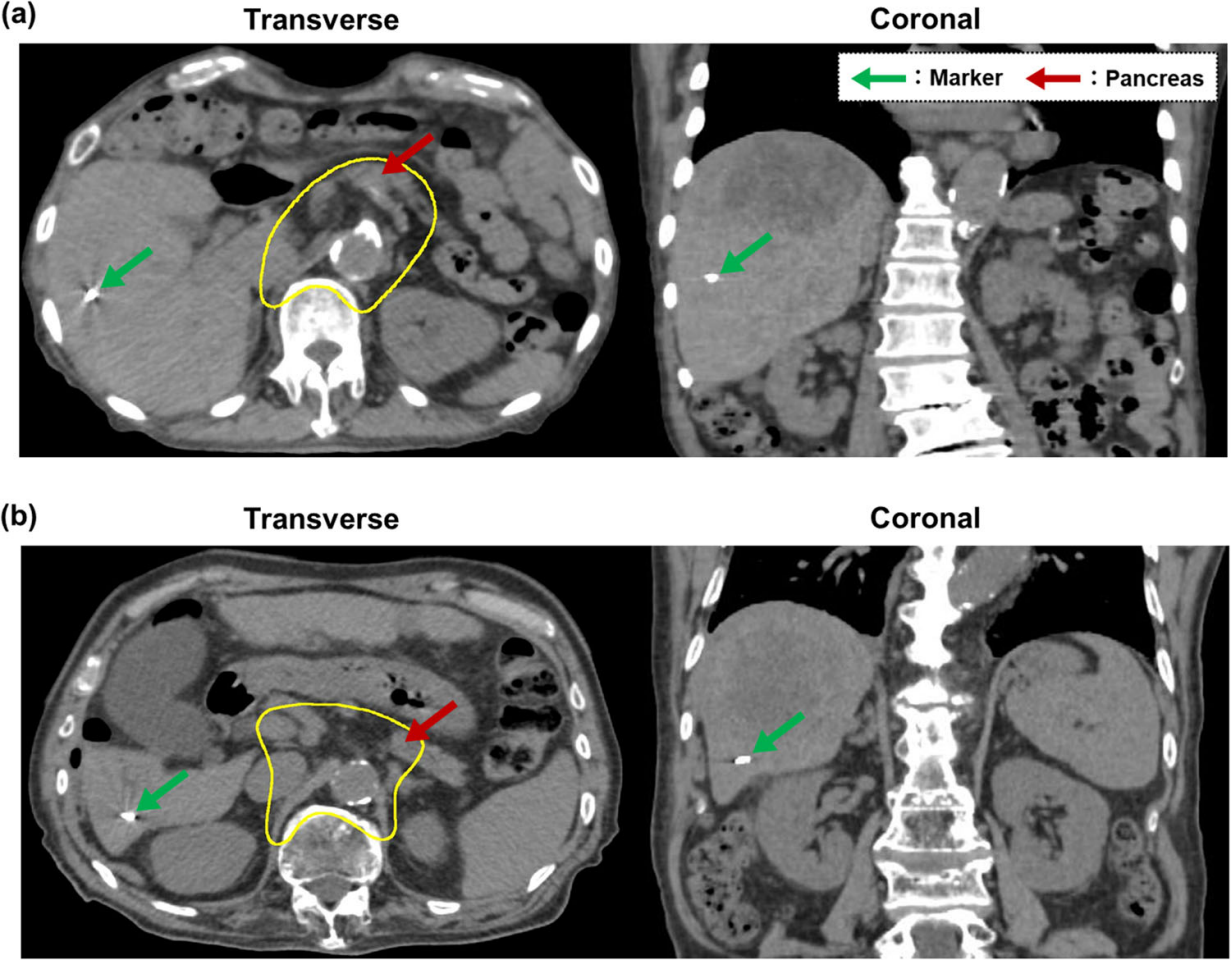
Location of the fiducial markers in the liver. Fiducial markers inserted in (a) S5 and (b) S6 are shown on the axial (left) and coronal (right) computed tomography (CT) images. Green and red arrows indicate the fiducial marker and pancreas, respectively. The yellow contour indicates the planning target volume (PTV).

A total of 20 consecutive patients who underwent scanning CIRT for liver cancer at our institution between July 2018 and June 2024 were included. Two Gold Anchor markers (Naslund Medical AB, Huddinge, Sweden) were implanted in S5 or S6 and in the upper part of the liver tumor away from the pancreas (Table 1, Figure 1). Considering radiation exposure and patient condition, irCT images were acquired at least three times during the treatment of 12 fractions. The irCT scanner used was the same as pCT scanner (Aquilion LB, Canon Medical Systems Corporation, Tochigi, Japan).^24^ The imaging and reconstruction protocols were identical for pCT and irCT scans, and the scan range for the irCT scan was the same as that for the pCT scan. This study was conducted following the guidelines of the Declaration of Helsinki and was approved by the Institutional Review Board of participating institutions. All patients provided written informed consent, and data were anonymized for the analyses.

**TABLE 1:** Patient characteristics.

Patient	Age	Sex	Position of the fiducial marker used in marker matching^*^	Number of in‐room CT scans
1	82	Male	S6	3
2	85	Female	S6	5
3	87	Male	S6	3
4	75	Male	S5	3
5	76	Male	S6	3
6	73	Female	S6	3
7	70	Male	S5	3
8	82	Female	S5	4
9	85	Male	S6	3
10	91	Male	S6	3
11	88	Male	S6	3
12	87	Male	S6	3
13	73	Male	S6	3
14	64	Male	S5	3
15	73	Male	S5	3
16	81	Male	S6	3
17	50	Female	S6	3
18	81	Male	S5	3
19	58	Male	S6	3
20	75	Male	S6	3

Abbreviations: CT = computed tomography; S5/S6 = segment 5/6.

* Hepatic segment based on Couinaud classification.

### 2.3 Initial plan

#### 2.3.1 Contouring

All patients were immobilized using a BlueBAG system (Elekta AB, Stockholm, Sweden) and Shellfitter (Kuraray, Tokyo, Japan) on the treatment couch. Patients were instructed to fast for 5 h before pCT scans or treatment and received enemas if they had not defecated within the preceding 24 h. The pCT images obtained during breath‐hold at maximum exhalation (pCT_50%_ images) were acquired using the pCT scanner.

Contouring was performed using MIM Maestro software (Version 7.3.5, MIM Software Inc., Cleveland, OH, USA). CTV and OARs, including the stomach, duodenum, small intestine, colon, skin, and spinal cord were delineated on pCT_50%_ images. The CTV was defined as the entire pancreas with a 5 mm isotropic margin, including regions such as the nerve plexus around the celiac and superior mesenteric arteries and the para‐aortic lymph nodes.^24^ The planning target volume (PTV) was generated by expanding the CTV by 3 mm in all directions, excluding regions overlapping with or adjacent to OARs. Typically, the PTV is set at 5 mm in our facility. This was determined by ICRU Report 62.^25^ However, in pancreatic cancer, because the CTV is surrounded by the gastrointestinal tract, the margin is set at 3 mm. In a 5 mm PTV, the setup margin is calculated based on the beam interlock condition, which is the emergency irradiation stop condition triggered when a beam misalignment occurs. Although the PTV margin was reduced to 3 mm, calculating the setup margin based on the trend value for irradiation accuracy, it is acceptable.

#### 2.3.2 Optimization of dose distributions

**FIGURE 2:**
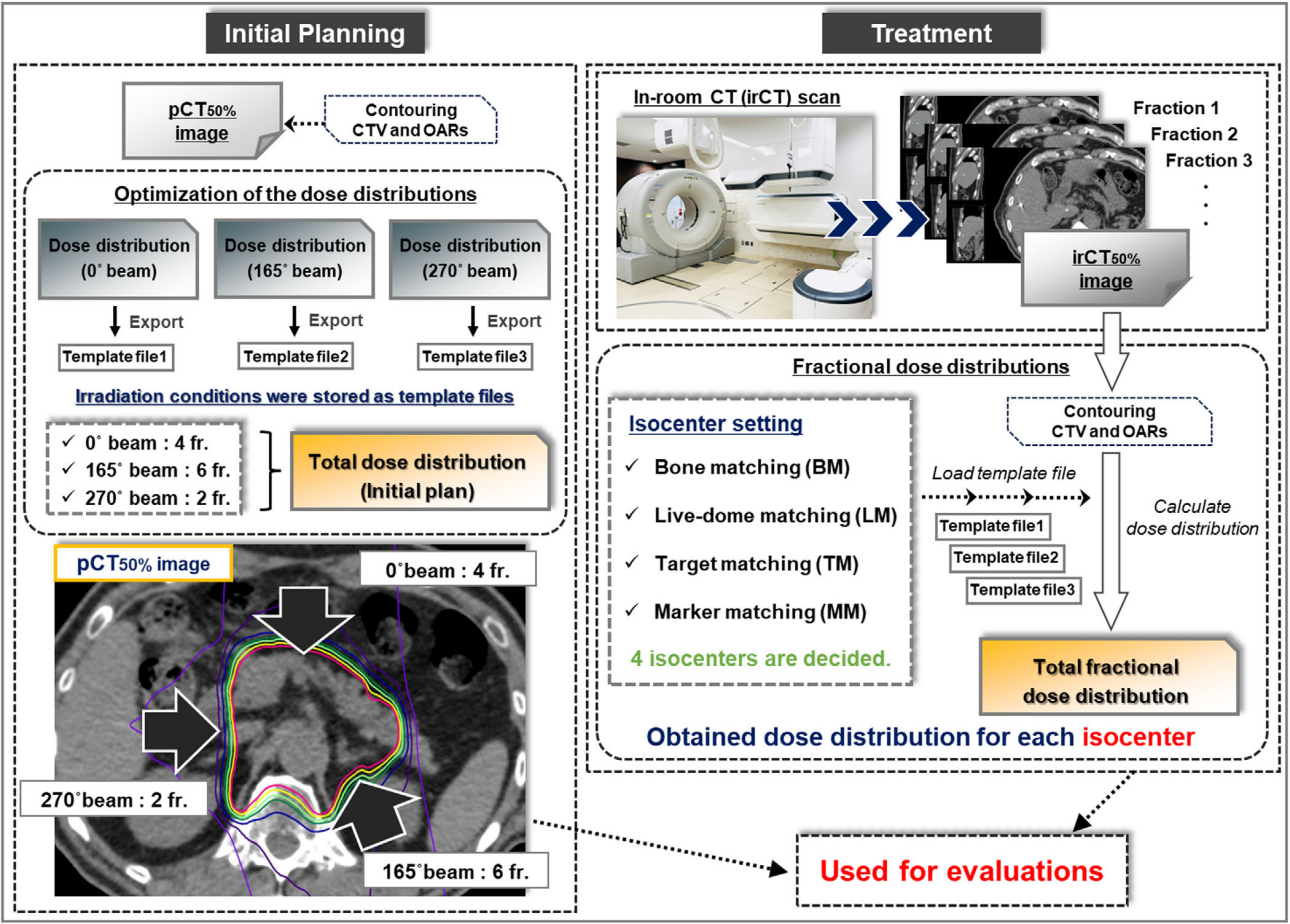
Evaluation workflow for each matching method.

The Monaco Treatment Planning System for carbon‐ions (Version 5.20, Elekta AB, Stockholm, Sweden) was used to optimize the dose distribution. The total RBE‐weighted absorbed dose was prescribed as 55.2 Gy/12 fractions.^26^ One beam was delivered per day during treatment. The microdosimetric kinetic model was used as the RBE model.^27^ Based on prior studies, the irradiation directions at gantry angles of 0°, 165°, and 270° were selected in the supine position, corresponding to 4, 6, and 2 fractions, respectively (Figure 2).^12^ The raster scanning method was used for the formation of the SOBP. This method combines SOBP formation in depth direction and lateral spot placement to produce a three‐dimensional uniform dose distribution. In treatment planning, the beam diameter was set to 3 mm (beam spot size), and the irradiation positions were arranged at 2 mm intervals on a plane perpendicular to the beam axis (spot spacing). The slice width was 2.5 mm in water equivalent depth (layer spacing), and the irradiation position (beam stop position) in the depth direction was set according to the PTV. Each irradiation position was irradiated with a narrow‐width SOBP of approximately 6 mm equivalent depth to water. Considering patient setup errors during treatment, the smearing function of Monaco was used. Smearing accounts for setup errors by reflecting them in the calculation of maximum and minimum beam ranges, resulting in a wider irradiation range in both the distal and proximal directions. Then, the mean value replacement method was adopted to prepare a robust plan to account for gastrointestinal gas.^12^ Dose distributions for each gantry angle were optimized to ensure that the PTV was covered by 95% of the prescribed dose (V95% = 100%) while prioritizing adherence to OAR dose constraints. The dose constraints for the gastrointestinal tract, skin, and spinal cord were set as follows: maximum dose to 2 cm^3^ (D_2cm3_) < 44 Gy, maximum dose to 0.5 cm^3^ (D_0.5cm3_) < 48 Gy, and maximum dose (D_max_) < 30 Gy, respectively. Dose constraints for OARs were evaluated based on the total dose. Following optimization, irradiation conditions were stored as templates for calculating fractional dose distributions for each treatment day (Figure 2).

### 2.4 Calculation of the fractional dose distribution

At our facility, daily patient setup is performed using 2D–3D bone matching (BM) based on frontal and lateral x‐ray images and the digitally reconstructed radiography derived from the pCT images. Additionally, irCT images obtained during breath‐hold at maximum exhalation (irCT_50%_ images) are periodically acquired without altering the patient setup determined by BM. In this study, BM, liver‐dome matching (LM), TM, and MM were reproduced using pCT_50%_ and irCT_50%_ images to determine the isocenter for each matching method. Based on the isocenter, fractional dose distributions for the three beam directions were calculated using the template without modification. CTV and OARs were also delineated on the irCT_50%_ images.

All registration processes were performed using MIM Maestro. BM and TM were implemented via rigid registration between pCT_50%_ and irCT_50%_ images. For BM, registration was performed using the bony structures surrounding the pancreas as the region of interest. For TM, the matching region was defined based on the delineated CTV. After registration, pCT_50%_ and irCT_50%_ images were visually inspected, and manual adjustments were applied in the left–right, anterior–posterior, and superior–inferior directions as needed. For LM, pCT_50%_ images were manually shifted in the left–right, anterior–posterior, and superior–inferior directions to align the diaphragm position with irCT_50%_ images. For MM, the fiducial marker closest to the pancreas among the two fiducial markers inserted into the liver (S5 or S6) was used for matching (Table 1). The pCT_50%_ images and irCT_50%_ images were manually shifted in the left–right, superior–inferior, and anterior–posterior directions so that the fiducial marker aligned. Finally, the isocenter from the initial planning, shifted to match each method's results, was transferred to the irCT_50%_ images　(BM, TM, LM, and MM isocenters). These isocenters were then used to calculate the fractional dose distributions.

### 2.5 Analysis of interfractional target motion

The interfractional target motion was calculated using pCT_50%_ and irCT_50%_ images. If no interfractional target motion exists, the BM and TM isocenters will coincide. Therefore, by analyzing the difference between the BM and TM isocenters in the pCT and irCT images, the interfractional target motion for each treatment day can be obtained. Rigid registrations with BM and TM between the pCT_50%_ and irCT_50%_ images were performed using MIM. As described in Section 2.4, BM and TM isocenters were determined by transferring the initial plan isocenter to the irCT_50%_ images according to each registration result. Subsequently, the displacements in the left–right, superior–inferior, and anterior–posterior directions (ΔPos(XYZ)) were obtained by calculating the difference between the BM and TM isocenters as follows:

(1) $$\Delta \mathrm{Pos}\left(\mathrm{XYZ}\right)=\mathrm{XY}Z_{\mathrm{Iso},\mathrm{TM}}-\mathrm{XY}Z_{\mathrm{Iso},\mathrm{BM}}$$

where XYZ_Iso,TM_​ and XYZ_Iso,BM_​ represent the coordinates of the TM and BM isocenters for each treatment day, respectively.

### 2.6 Correlation analysis between the motions of target and fiducial marker or liver‐dome

To quantitatively investigate the correlation between the motions of the target and the fiducial markers or liver‐dome, the displacements of the isocenter from the BM coordinates at TM, MM, and LM (ΔXYZ_match_) were obtained as follows:

(2) $$\Delta \mathrm{XY}Z_{\mathrm{match}}=\mathrm{XY}Z_{\mathrm{Iso},\mathrm{match}}-\mathrm{XY}Z_{\mathrm{Iso},\mathrm{BM}}$$

where XYZ_Iso,match_​ represents the coordinates of the isocenter determined by the TM, MM, or LM. Both XYZ_Iso,match_ and XYZ_Iso,BM_​ were obtained using the registration procedures described in Section 2.4. In this study, irCT_50%_ images acquired in clinical practice of liver CIRT were used. These irCT_50%_ images were acquired using various matching methods, such as BM, MM, and LM, resulting in differences in central coordinates among treatment days. To align the central coordinates among the irCT_50%_ images, the coordinate differences relative to the BM position were calculated. Subsequently, the displacements of the fiducial marker and liver‐dome were plotted against the displacement of the target, and motion correlations were evaluated.

### 2.7 CTV coverage and OAR dose evaluations

CTV V95% for the initial plan (CTV V95%_pCT50%_) and each treatment day (CTV V95%_irCT50%_) were analyzed for all beams (the total dose) and single beams for each gantry angle (dose per single beam). Furthermore, OAR doses for the initial plan (D_OAR,pCT50%_) and each treatment day (D_OAR,irCT50%_) were analyzed based on the total dose. The isocenter for each matching method was determined for each treatment day. As presented in Table 1, irCT_50%_ images were acquired 3–5 times per patient. Target coverage and OAR dose evaluations were performed for all irCT_50%_ images. Thus, 3–5 evaluations were performed per patient. Finally, the variations from the initially planned CTV V95% (ΔCTV V95%) and OAR dose (ΔD_OAR_) were evaluated as follows:

(3) $$\Delta \mathrm{CTV}\;V95\%=\mathrm{CTV}\;V95{\%}_{\mathrm{irCT}50\%}-\mathrm{CTV}\;V95{\%}_{\mathrm{pCT}50\%}$$

(4) $$\Delta D_{\mathrm{OAR}}=D_{\mathrm{OAR},\mathrm{irCT}50\%}-D_{\mathrm{OAR},\mathrm{pCT}50\%}$$

OARs included the gastrointestinal tract, skin, and spinal cord, and the doses D_2cm3_, D_0.5cm3_, and D_max_ were evaluated.

### 2.8 Statistical analysis

The interfractional target motion in the three directions (ΔPos(XYZ)) was evaluated. As this data were not normally distributed, the Friedman test was used to compare significant differences among the three groups. To account for multiple comparisons, the significance level was adjusted by dividing it by three using the Bonferroni method.

The correlation between the motions of the target and the fiducial marker or liver‐dome was also evaluated. As these data were not normally distributed, Spearman's rank correlation coefficient (*ρ*) was used to evaluate the correlation.

ΔCTV V95% and ΔD_OAR_ were evaluated using four different matching methods on identical irCT_50%_ images acquired during the treatment period. The Friedman test, suitable for non‐normally distributed data, was used to compare significant differences among the four groups. To account for the multiple comparisons required, *p*‐values were adjusted by dividing by four using the Bonferroni method.

All statistical analyses were performed using SPSS (IBM SPSS Statistics, Version 29.0, IBM Inc., Armonk, NY, USA). For all tests, the initial significance level was set at *p* < 0.05, with Bonferroni corrections applied for multiple comparisons as described above.

## 3 RESULTS

### 3.1 Interfractional target motion

**FIGURE 3:**
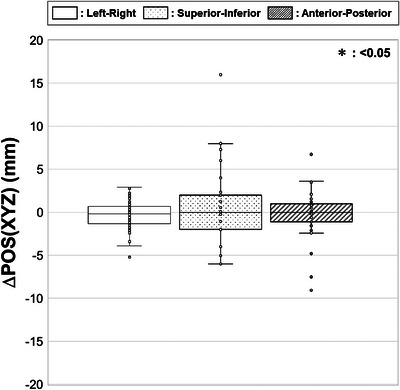
Interfractional target motion after bone matching. Boxplots showing median, first and third quartiles, maximum, minimum, and outliers for displacements from initial plan (ΔPOS(XYZ)) in left–right, superior–inferior, and anterior–posterior directions.

Figure 3 illustrates the interfractional target motion in the left–right, superior–inferior, and anterior–posterior directions. Although no statistically significant differences were observed among the directions, the displacement was greatest in the superior–inferior direction.

### 3.2 Correlations between the motions of target and fiducial marker or liver‐dome

**FIGURE 4:**
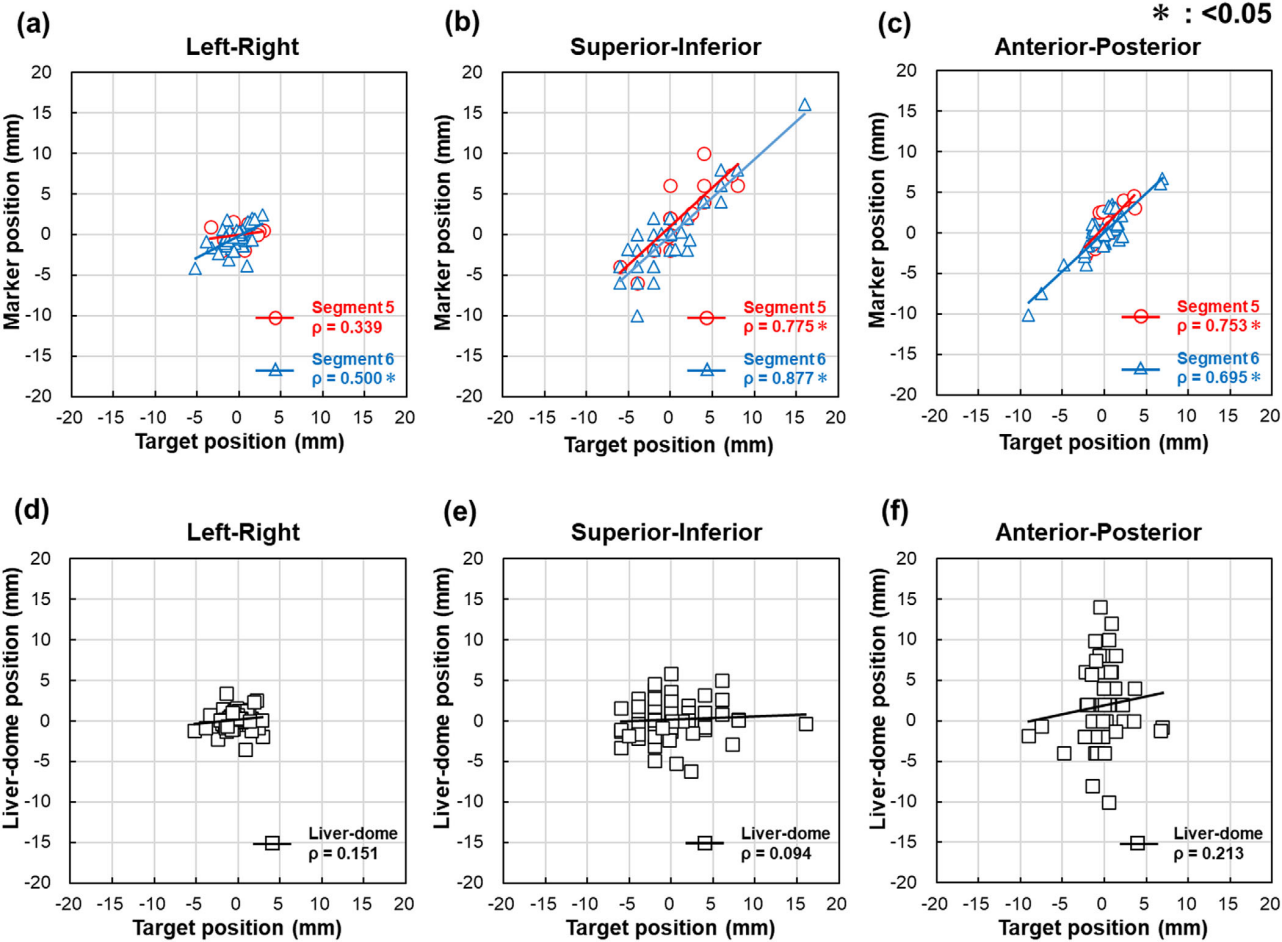
The correlation between motions of the target and the fiducial marker or liver‐dome. Correlation between the target motion and the motions of fiducial markers (Segment 5: red circles, Segment 6: blue triangles) in (a) left–right, (b) superior–inferior, and (c) anterior–posterior directions, and the liver‐dome (black squares) in (d) left–right, (e) superior–inferior, and (f) anterior–posterior directions. The correlation coefficient (*ρ*) is shown in each panel. Statistically significant differences are indicated by an asterisk (*).

Figure 4 illustrates the correlations between the motions of the target and the fiducial marker or liver‐dome. Strong correlations were observed in the superior–inferior and anterior–posterior directions, whereas correlations in the left–right direction were low. In contrast, no correlation was observed between the motions of the target and the liver‐dome.

### 3.3 CTV coverage

**FIGURE 5:**
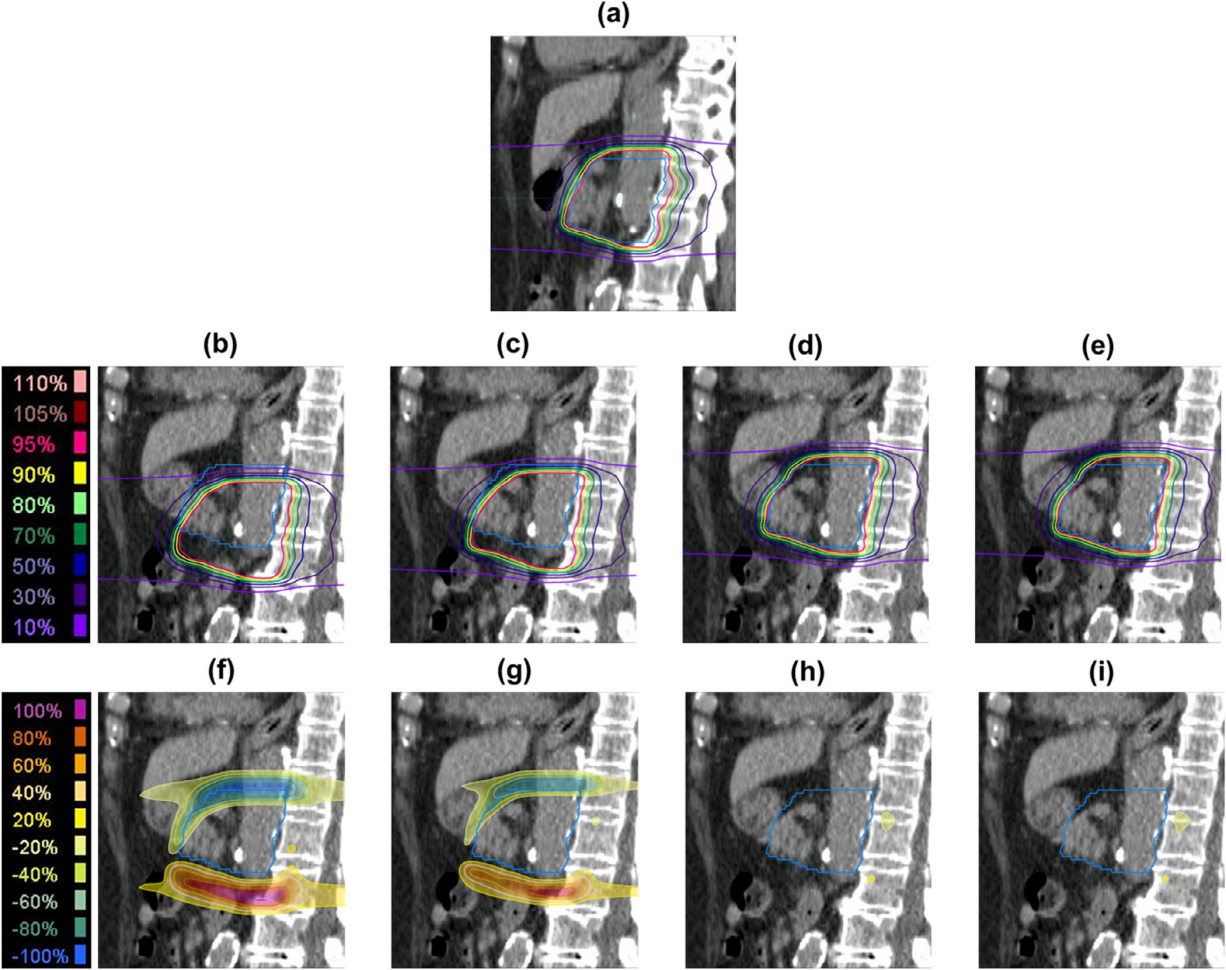
Example of dose distributions for each matching method. Dose distributions for (a) initial plan, (b) bone matching, (c) liver‐dome matching, (d) target matching, and (e) marker matching. The dose difference maps for (f) bone matching, (g) liver‐dome matching, (h) target matching, and (i) marker matching, each relative to (a) the initial plan. The blue contour line indicates the clinical target volume.

**FIGURE 6:**
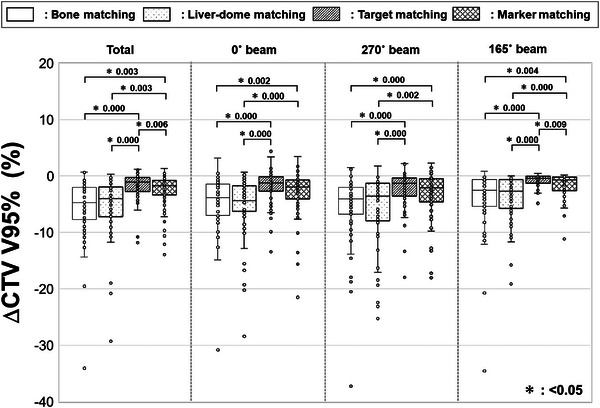
Variation in the clinical target volume coverage from the initial plan (ΔCTV V95%) for each matching method. Box‐and‐whisker plots display the median, first quartile, third quartile, maximum, minimum, and outliers of ΔCTV V95% for bone, liver‐dome, target, and marker matching across the total dose and every single beam. Statistically significant differences are indicated by an asterisk (*).

Figure 5 illustrates an example of the dose distributions and dose difference maps during the initial plan and for each matching method. Compared to BM and LM, TM and MM demonstrate an improvement in target coverage. Table 2 represents the median CTV V95%_irCT50%_ for each matching method, including evaluations for single beam dose at three gantry angles and total beam dose. Figure 6 shows the boxplot of ΔCTV V95% for each matching method. The median ΔCTV V95%_irCT50%_ for total beam dose in BM, LM, TM, and MM were −4.68% (range, −34.08%–0.72%), −3.97% (range, −29.27%–0.32%), −1.07% (range, −11.81%–1.24%), and −1.69% (range, −13.93%–1.36%), respectively. TM and MM significantly improved target coverage compared to BM (*p* < 0.05). LM was equivalent to BM. A similar trend was observed in evaluations for single beams.

**TABLE 2:** Target coverage (CTV V95%).

	CTV V95%_pCT50%_ (%)	CTV V95%_irCT50%_ (%)			
Gantry Angle	Initial plan	BM	LM	TM	MM
0 ° beam (18.4 Gy)	97.89 (93.93–99.5)	93.20 (66.48–99.06)	93.96 (69.31–99.66)	96.06 (84.31–99.84)	95.63 (76.23–99.54)
165 ° beam (27.6 Gy)	98.33 (91.03–99.96)	92.90 (62.40–98.87)	93.04 (65.78–98.92)	96.20 (77.48–99.96)	96.15 (73.81–99.91)
270 ° beam (9.2 Gy)	99.85 (99.29–99.99)	97.40 (65.27–99.97)	97.17 (80.65–99.83)	99.32 (95.16–99.98)	98.89 (88.37–99.97)
3‐beam total (55.2 Gy)	98.89 (96.88–99.96)	94.27 (64.80–98.89)	94.49 (67.62–99.83)	97.57 (85.07–99.90)	97.14 (81.83–99.70)

*Notes*: The upper row of the table indicates the median value. Values in parentheses indicate minimum to maximum values. A clinically acceptable value is defined as V95% ≥ 95%.

Abbreviations: BM = bone matching; CTV V95%_pCT50%_ = CTV V95% in initial plan; CTV V95%_irCT50%_ = CTV V95% on each treatment day; Gy = relative biological effectiveness‐weighted absorbed dose; LM = liver‐dome matching; MM = marker matching; TM = target matching.

### 3.4 OAR dose

**FIGURE 7:**
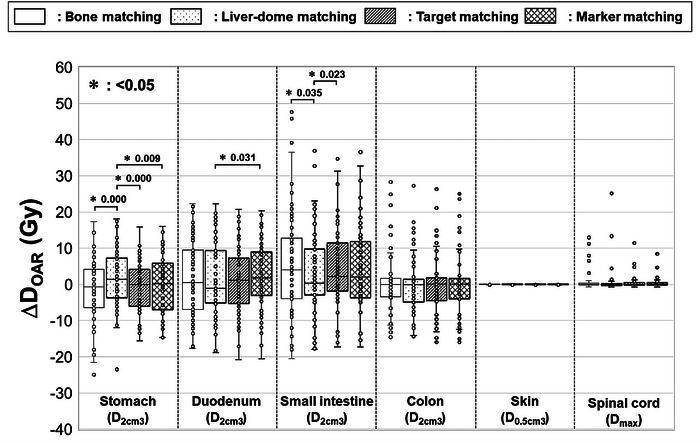
Variation in the OAR dose from the initial plan (ΔD_OAR_) for each matching method. Box‐and‐whisker plots display the median, first quartile, third quartile, maximum, minimum, and outliers of ΔD_OAR_ for bone, liver‐dome, target, and marker matching across the total dose. Statistically significant differences are indicated by an asterisk (*). D_2cm3_ = maximum dose that covered 2 cm^3^; D_0.5cm3_ = maximum dose that covered 0.5 cm^3^; D_max_ = maximum dose; Gy = relative biological effectiveness‐weighted absorbed dose.

Table 3 represents D_OAR,irCT50%_ for each matching method. Although some individual cases showed temporary exceedance of dose constraints when evaluated on a specific treatment day. In this study, OAR doses were evaluated using the total dose distribution recalculated on the irCT_50%_ images acquired on each treatment day. In actual clinical practice, only one beam is irradiated per day; however, when evaluated over the entire treatment course (12 fractions), OAR doses remained within the dose constraints. Figure 7 shows the boxplot of ΔD_OAR_ for each matching method. Minimal dose variation was observed in the skin and spinal cord across all matching techniques. In the gastrointestinal tract, statistically significant differences were detected between some matching method pairs (*p* < 0.05); however, no consistent differences were observed overall among the four methods.

**TABLE 3:** Organs‐at‐risk (OAR) dose.

			D_OAR,pCT50%_ (Gy)	D_OAR,irCT50%_ (Gy)			
Organ	DVH.P	Constraints	Initial plan	BM	LM	TM	MM
Stomach	D_2cm3_	<44 Gy	36.79 (22.23–42.64)	35.46 (13.75–52.48)	38.03 (14.97–54.77)	35.83 (14.30–52.52)	36.62 (14.07–52.98)
Duodenum	D_2cm3_	<44 Gy	32.80 (16.11–38.17)	34.00 (12.17–55.02)	31.32 (10.56–54.91)	33.32 (10.59–53.82)	34.85 (10.80–53.41)
Small intestine	D_2cm3_	<44 Gy	19.23 (0.52–40.07)	21.56 (0.84–55.04)	19.83 (0.54–53.01)	24.41 (0.92–49.68)	20.08 (0.72–54.74)
Colon	D_2cm3_	<44 Gy	15.09 (5.94–38.00)	15.15 (4.97–52.40)	14.52 (4.82–52.34)	15.45 (5.09–52.92)	15.25 (5.11–52.70)
Skin	D_0.5cm3_	<48 Gy	16.27 (14.49–18.73)	16.23 (14.49–18.76)	16.23 (14.49–18.75)	16.23 (14.50–18.76)	16.23 (14.49–18.73)
Spinal cord	D_max_	<30 Gy	18.29 (16.39–20.53)	18.67 (16.27–30.20)	18.71 (16.00–42.67)	18.75 (16.12–29.48)	18.69 (16.11–26.59)

*Notes*: The upper row of the table indicates the median value. Values in parentheses indicate minimum to maximum values.

Abbreviations: BM = bone matching; DVHP = dose volume histogram parameter; D_OAR,pCT50%_ = OAR dose in initial plan; D_OAR,irCT50%_ = OAR dose on each treatment day; D_2cm3_ = maximum dose that covered 2 cm^3^; D_0.5cm3_ = maximum dose that covered 0.5 cm^3^; D_max_ = maximum dose; Gy = relative biological effectiveness‐weighted absorbed dose; MM = marker matching; LM = liver‐dome matching; TM = target matching.

## 4 DISCUSSION

This study evaluated the correlations between the motions of the target and the S5 or S6 intrahepatic fiducial marker, as well as target coverage and OAR doses in pancreatic cancer CIRT using four setup strategies: BM, LM, TM, and MM. Consequently, a strong correlation was observed between the motions of the pancreatic target and the fiducial marker implanted in the S5 or S6 hepatic region. Furthermore, MM significantly improved target coverage compared to BM and LM. Fiducial markers implanted caudally in the liver were effective in correcting interfractional target motion for pancreatic cancer. The MM method proposed in this study can be applied not only to CIRT but also to photon and proton therapy.

In LM, no improvement in target coverage was observed; its performance was comparable to that of BM. The correlation between the motions of the target and the liver‐dome was also low. This is likely due to differences in the motion characteristics of the pancreas and the liver‐dome. While the pancreas moves with respiration, the liver‐dome undergoes shape deformation caused by cyclical relaxation and contraction. These findings are consistent with those of Itabashi et al., who reported distinct differences in motion patterns between pancreatic tumors and the liver‐dome.^28^ TM significantly improved target coverage compared to MM, as it directly corrects interfractional target motion using the pancreas as the reference. In MM, interfractional target motion was corrected indirectly using a fiducial marker implanted in the liver adjacent to the pancreas. This anatomical separation may have introduced motion correlation errors.

**FIGURE 8:**
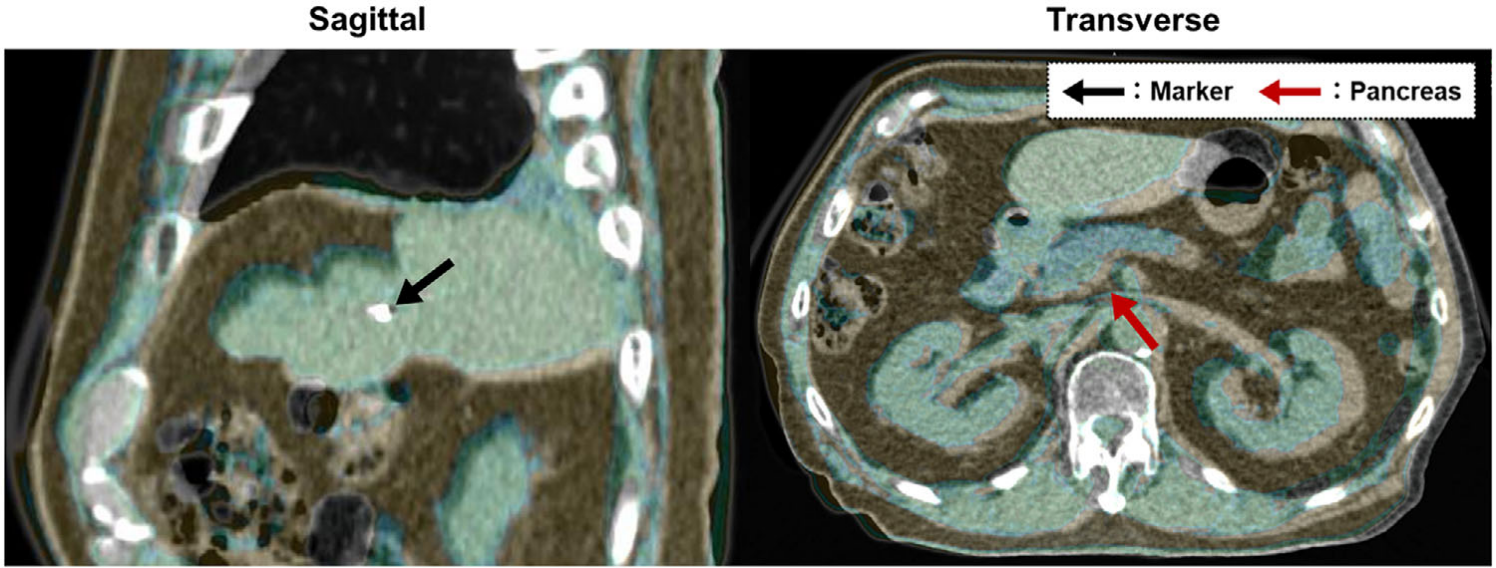
Example of overlaid image of pCT_50%_ and irCT_50%_ images with marker matching method. The gray and green areas indicate pCT_50%_ images and irCT_50%_ images, respectively. Sagittal image is shown on the left, and axial image on the right. The black and red arrows indicate the positions of the fiducial marker with marker matching and the pancreas, respectively.

Correlation analysis between the motions of the target and the fiducial marker showed, a strong correlation in the superior–inferior and anterior–posterior directions; however, the correlation was low in the left–right direction (Figure 4). Figure 8 shows an example of an overlaid image of the pCT_50%_ and irCT_50%_ images with MM, where the position of the pancreas was shifted to the patient's right side. These results suggest that target coverage decreased in MM compared with TM because of insufficient correction in the left–right direction. However, considering the relatively small differences in target coverage between MM and TM, as well as the significant improvement of MM over BM, the MM approach proposed in this study can be considered an effective motion correction method.

Patients with hepatic CIRT included in this study had two markers inserted into the liver. These markers were placed for liver cancer treatment; therefore, one was inserted near the pancreas and in the upper part of the liver tumor away from the pancreas. Consequently, only the fiducial marker closest to the pancreas was used for MM. Interfractional target motion was corrected only in the left–right, anterior–posterior, and superior–inferior directions because the pancreas and liver are separate organs, and rotation detected using a marker inserted far from the pancreas may not necessarily reflect pancreatic motion. The results of this study showed that interfractional target motion can be corrected using a single marker; however, inserting two markers close to the pancreas would allow rotational correction in the pancreatic region, potentially improving the accuracy of interfractional target motion correction.

The variation in OAR dose from the initial plan was also observed. These were primarily attributed to interfractional OAR motion and shape changes resulting from fluctuations in gastrointestinal gas volume.^29^, ^30^ Although statistically significant dose differences were detected in some OARs across certain matching methods, no consistent differences between matching methods were observed. Currently, BM is the standard clinical setup method at our facility. Based on the present findings and dose‐volume histogram parameter analysis, adverse event rates with MM are expected to be similar.

With the ongoing development of advanced techniques such as dose escalation and LET optimization to improve local control rates in pancreatic cancer,^31^, ^32^, ^33^, ^34^, ^35^, ^36^, ^37^ higher precision in dose delivery is increasingly required. The MM method proposed in this study significantly improved target coverage compared to the conventional BM method while maintaining OAR doses within acceptable constraints, suggesting its viability as a clinically applicable strategy. Moreover, MM enhances treatment throughput compared to TM, as it enables rapid confirmation of tumor position and compensation for interfractional motion through radiography during patient setup, unlike TM, which requires time‐consuming irCT scan. Additionally, MM allows for real‐time tumor localization using x‐ray fluoroscopy during irradiation. Tumor motion caused by breathing during irradiation is most pronounced in the superior–inferior direction. Although alignment errors remain in the left–right direction, the motion of the fiducial marker in the superior–inferior direction closely reflects the motion of the pancreas. While further verification is needed, irradiation using marker tracking may be feasible in the future. MM may facilitate high‐precision treatment while preserving throughput and workflow efficiency, potentially contributing to improved treatment outcomes.

For clinical implementation, verifying the correlation between the motions of the pancreas and the fiducial marker in the S5 or S6 region at treatment planning is essential on a patient‐specific basis. At our facility, patients undergoing pancreatic cancer CIRT receive a 4D‐CT scan during treatment planning.^12^ For each respiratory phase, the correlation can be evaluated by comparing the motions of the pancreas and the fiducial marker. If the correlation is insufficient, or if displacement of the irradiation position is observed during irCT image confirmation before the first irradiation, the irradiation position should be determined using TM. Furthermore, the shellfitter used at our facility not only fixes the patient's position but also suppresses breathing through compression. Variations in the shellfitter's compression strength can lead to variations in organ motion; therefore, it is necessary to ensure reproducibility so that the compression during treatment planning CT matches that during treatment.

The study's primary limitation was the limited sample size of only 20 cases. Although the data used in the analysis are adequate for initial analysis, a larger sample size could improve statistical power and generalizability. Second, this study focused exclusively on cases with fiducial markers in S5 or S6. If the fiducial marker is implanted in regions other than the S5 or S6, further validation may be required. Finally, the interfractional motion of both tumors and OARs may vary depending on patient immobilization and facility‐specific setup protocols. If MM is implemented clinically, its effectiveness should be evaluated under site‐specific conditions using irCT or other imaging modalities.

## 5 CONCLUSION

This study investigated the correction of interfractional target motion in pancreatic cancer CIRT using fiducial markers implanted in the liver regions S5 or S6. The MM approach significantly improved target coverage, achieving results comparable to those of TM. However, variation in target coverage was still observed with MM, suggesting the need for periodic anatomical assessment using irCT or alternative imaging methods. In pancreatic cancer CIRT, both interfractional target motion and anatomical changes caused by gastrointestinal gas or body shape variations must be managed to ensure optimal target coverage.

## AUTHOR CONTRIBUTIONS

**Junya Nagata**: Data curation; formal analysis; investigation; methodology; writing—original draft. **Yohsuke Kusano**: Conceptualization; funding acquisition; methodology; supervision; writing—original draft. **Masataka Komori**: Supervision. **Yoshiki Takayama**: Writing—review and editing. **Shogo Kurokawa**: Writing—review and editing. **Atsushi Ito**: Writing—review and editing. **Tadashi Kamada**: Writing—review and editing. **Daisaku Yoshida**: Writing—review and editing. **Shohei Kawashiro**: Writing—review and editing. **Hiroyuki Katoh**: Writing—original draft. All authors were involved in the data analysis, critically revised the report, commented on drafts of the manuscript, and approved the final report.

## CONFLICT OF INTEREST STATEMENT

Dr. Hiroyuki Katoh, Dr. Daisaku Yoshida, and Dr. Yohsuke Kusano received research funding from Toshiba Energy Systems and Solutions Corporation.

## ETHICS STATEMENT

This study was conducted following the guidelines of the Declaration of Helsinki and was approved by the Institutional Review Board of Kanagawa Cancer Center (Approval Number: 2024eki‐8) and Nagoya University (Approval Number: 24–305). All patients provided written informed consent, and data were anonymized for the analyses.

## Data Availability

The datasets used and/or analyzed during the current study can be obtained from the corresponding author upon reasonable request.
